# How to Draw the Line Between Health and Disease? Start with Suffering

**DOI:** 10.1007/s10728-021-00434-0

**Published:** 2021-04-29

**Authors:** Bjørn Hofmann

**Affiliations:** 1 Department for the Health Sciences, Norwegian University of Science and Technology (NTNU), Gjøvik, Norway; 2 Centre of Medical Ethics, University of Oslo, Blindern, PO Box 1130, N-0318 Oslo, Norway

**Keywords:** Health, Disease, Naturalist, Normativist, Demarcation, Dysfunction

## Abstract

How can we draw the line between health and disease? This crucial question of demarcation has immense practical implications and has troubled scholars for ages. The question will be addressed in three steps. First, I will present an important contribution by Rogers and Walker who argue forcefully that no line can be drawn between health and disease. However, a closer analysis of their argument reveals that a line-drawing problem for disease-related features does not necessarily imply a line-drawing problem for disease as such. The second step analyzes some alternative approaches to drawing the line between health and disease. While these approaches do not provide full answers to the question, they indicate that the line-drawing question should not be dismissed too hastily. The third step investigates whether the line-drawing problem can find its solution in the concept of suffering. In particular, I investigate whether returning to the origin of medicine, with the primary and ultimate goal of reducing suffering, may provide sources of demarcation between health and disease. In fact, the reason why we pay attention to particular phenomena as characteristics of disease, consider certain processes to be relevant, and specific functions are classified as dys-functions, is that they are related to suffering. Accordingly, using suffering as a criterion of demarcation between health and disease may hinder a wide range of challenges with modern medicine, such as unwarranted expansion of disease, overdiagnosis, overtreatment, and medicalization.

## Introduction

What falls under the concept of disease has expanded significantly over the years [15, 30, 39, 42, 60–62, 64–66, 83]. While this expansion has enabled helping more people, it has also drawn attention to its negative implication, such as overdiagnosis, overtreatment, and medicalization [10, 44, 52, 58]. It is currently widely asked whether we are doing too much? [9, 24, 50, 63, 67, 68, 72, 92] At the heart of this challenge lies the problem of drawing the line between health and disease. How can we demarcate what is disease from what is not? The answer to this question is crucial for persons, patients, health professionals, health policy makers, and for society at large. The line between health and disease has demarcated what deserves medical attention and what does not as well as what is a subject matter for health care and not. It has puzzled scholars for ages.

In a comprehensive recent article Wendy Rogers and Mary Walker point out how difficult this line-drawing problem is. They use examples from disease indicators, risk factors, biological processes, and paraclinical measures to underscore their point and argue that a distinct boundary between health and disease cannot be provided [74]. But without a line between health and disease, everything may become an issue for health care, undermining a traditional resource for demarcation.

Although I agree with Rogers and Walker that it is difficult to draw the line between health and disease [32, 37], I think the last word may not be said. The field deserves closer scrutiny. First, the arguments for unclear distinctions, misted transitions, vague concepts, or blurred boundaries between health and disease may not be as convincing as thought by first sight. Second, there may be theories of health or disease that provide demarcation. Third, we may be looking for the demarcation line in the wrong place.

Accordingly, the objective of this study is to scrutinize a forceful argument that it is impossible to draw a line between health and disease. To set the scene, I will present and discuss the argument by Rogers and Walker and especially their claim that we cannot draw the line between disease and non-disease based on the concept of dys-function [74]. The analysis of Rogers and Walker’s arguments reveals some alternatives for drawing a line between health and disease on a functional level, which are briefly discussed in the second part. In the third part, I scrutinize whether we are looking for a line in the wrong place, and that we need to go back to the moral foundation of medicine: human suffering. Those phenomena that cannot be clearly related to the suffering experienced by the individual and recognized by others and that can be conceptualized, detected, and treated or prevented by health professionals, do not count as disease.

Importantly, what motivates the laborious line-searching endeavor is the need to delimit the tasks of health care in times with expanding possibilities and limited available resources.

## No Lines in Functional Conceptions of Disease

Dysfunction is a key criterion for disease [4, 6, 8]. Hence, showing that dysfunction cannot be clearly delimited from function is one forceful way to argue that there is no line between health and disease. This is the way of argument Rogers and Walker use to conclude that «dysfunction cannot provide a categorical boundary between disease and non-disease»[74]. In doing so, they refer to traditional theories of health and disease listed in Table 1.

**Table 1:** Brief overview over some traditional theories of health and disease and their accounts of function

Theory of health and disease	Short description	Reference
Biostatistical theory (BST)	BST is based on a statistical account of function («normal function»)	Boorse, [7]
Harmful dysfunction account (HDA)	HAD refers to an evolutionary (as intended by nature) account of function	Wakefield, [84]
Function-and-negative-consequences account (FNC)	Dysfunction is a disease only when it has negative consequences and is statistically below some mean (a «fix» of the statistical account of function)	Schwartz, [74]

With reference to these theories, they use three types of examples to illustrate that a line cannot be drawn between health and disease based on dysfunction. The first is that there is no natural line to be found on the level of indicators and risk-factors, such as glucose levels and hypertension. The second is that there is no line to be traced in physiological processes, such as in cancer growth. The third is that even in incontrovertible cases, such as infections, no lines are to be identified in terms of measures of dysfunction either.

Accordingly, if you cannot draw a sharp line between function and dysfunction, then you cannot draw the line between health and disease, which has important implications for defining and delimiting the tasks of health care. Let us investigate their argument in more detail in order to see what is at stake.

### Drawing the Line Between Indicators and Functions

Rogers and Walker use blood pressure, glucose levels (diabetes) and BMI (obesity) as examples of difficulties with line-drawing. «The changing nature of these thresholds suggests that there is no incontrovertible point at which function switches to dysfunction.»[74] As we cannot draw the line on such thresholds, we cannot draw the line on functions or demarcate disease from health.

However, this argument does no heavy lifting. Blood pressure, glucose levels, or BMI are not functions, but rather (remote) indicators of functions [40]. Blood pressure as such has no direct function in the body but may indicate the function of the heart, blood vessels, and tissues. Hence, the line-drawing problem discussed here is not one of the function of the heart (or the tissues), the metabolism, or the physical performance as such. It is the line-drawing problem of risk factors and indicators of such functions.

Hence, it can be argued that there is a mix-up of indicators (or indicative phenomena) and functions [39, 40]. In an indirect manner Rogers and Walker acknowledge this when they write about variables, e.g., how «lower levels of the relevant variables [such as blood pressure, glucose level, and BMI] are classified as normal or healthy» [74]. Nonetheless, equaling «variables» and functions appears to be a confusion of indicators with what they indicate, i.e. their function. In semiotic terms, there appears to be confusion between the signifier and what is signified.

Rogers and Walker are perfectly right that «…hypertension could plausibly be 139/89 or 141/91 mmHg … The increments that separate having a disease and not having it are so small that the exact position of the line demarcating disease from non-disease—the diagnostic threshold—seems arbitrary.» [74]. However, it is not clear that the line-drawing problem of indicators is the same as the line-drawing problem of function—or disease for that matter.

While it is fully reasonable to believe that if functional line-drawing is difficult, so is line-drawing of functional indicators. However, Rogers and Walkers point is the opposite. They argue that functional line-drawing is difficult by referring to the line-drawing problem of indicators (of functions). For making the argument work, they would have to give an account for the one-to-one (or all-including) relationship between indicators and functions. However, the relationship between blood pressure and cardiovascular function is complex. Hence, while they may be perfectly right about the line-drawing about functions, they cannot use indicators to show this without giving a much more detailed account of the relationship between indicators and functions. To some extent they do elsewhere [73], but their outline is too brief to convince us that the line-drawing problem is unsurmountable.

This point comes out even clearer when they write: «The lack of a clear boundary is also indicated by the way that these conditions morph seamlessly into risk factors (pre-hypertension, pre-diabetes, overweight, etc.), suggesting that there are only shades of grey rather than quantifiable functional deficits between, for example, healthy blood pressure, pre-hypertension, and overt hypertension» [74]. They seem to take for granted that hypertension and obesity are dys-functions and not risk-factors. No doubt, these conditions have become risk-based diseases [79, 80], but it is not clear that they are diseases in merit of dys-function, which is the issue when you want to argue that we have a line-drawing problem in functional accounts of disease. Put bluntly, the line-drawing problem in risk for disease does not (directly) account for a line-drawing problem in dysfunction, as the relationship between risk-factors and dysfunction may be complex, weak, and probabilistic.

Hence, while I agree with the claim that «it seems implausible to claim that the presence of dysfunction can distinguish between health and disease regarding these disorders defined in terms of continuous variables and their associated risks» [74], I do not think that this can be shown by referring to the line-drawing problems of indicators or risk-factors—at least not without a series of further qualifications about the relationship between functions and such factors.

### Drawing the Line Between Process and Dysfunction

While it is easy to acknowledge that there is a line-drawing problem with spectrum type diseases, it may not be so for other types of disease, such as cancers. However, Rogers and Walker do a very good job in pointing out that cancers are not as easily defined as one may think. It is difficult to draw the line between health and disease based on typological, morphological, genetic, or behavioral characteristics of cancer as «cancerous growth occurs on a spectrum of deregulation. There may be cells with genetic markers but no subsequent abnormalities in growth, abnormal but nonmalignant cell growth, malignant-looking tumors that do not metastasize, all the way through to cancer with distant metastases.» [74].

Again, I think Rogers and Walker are very successful in arguing that it is difficult to draw the line between health and disease in various physiological processes, such as cell or tissue modification. However, it is far from obvious that the line-drawing problem of processes correspond to line-drawing problems of functions (and thereby of disease). Admittedly, by moving from (the line-drawing problems in) indicators and risk factors to (the line-drawing problems in) processes, they get closer to their core issue, i.e., (dys)function. However, it is still not clear that change in process is identical with disease-defining dysfunction. There are lots of processual changes that are highly functional, e.g., during pregnancy and birth.

However, as Rogers and Walker themselves succinctly point out, cellular change of function is not sufficient for demarcating disease from health. Hence, it becomes a question of functional level. When they argue that it is not sufficient to refer to morphological change or to «rely on genetic markers to identify dysfunction,» this may not be the level of change that is relevant for disease-demarcating dysfunction. There may be all sorts of complex (inter)acting underlying processes going on without being relevant to the overall function of vital organs or processes.

As Rogers and Walker themselves point out: «Searching for the dysfunction that marks health from disease in the case of cancer triggers an infinite regress down to molecular variations, none of which might be the sole cancer-causing dysfunction. It seems there may be no specific dysfunction that picks out cancer. Not all genetic mutations lead to abnormal cell growth; not all cases of atypia progress to dysplasia, and so forth.» [74]

I think that Rogers and Walker are perfectly right when they point out that: «as our understanding of cancer becomes more sophisticated, it becomes increasingly difficult to pinpoint the relevant dysfunction, and hence determine where any line should be drawn between the presence or absence of cancer.» [74] However, showing that the line between health and disease cannot be drawn in terms of low-level (indicative) processes does not amount to a knock down argument that the line cannot be drawn on some higher (functional) level. In order for the argument to do such work one has to assure that there is a known and predictable connection between the low and high-level function. However, Rogers and Walker convincingly have shown through their examples that such a relationship is not known.

Hence, the problem of drawing the line between health and disease on the level of processes does not warrant the conclusion that such a line cannot be drawn on other functional levels. There may well be a difference between ordinary processes and dysfunction, i.e., between physiology and pathology, on another level of function.

### Drawing the Line Between Measurements and Dysfunction

The third line of argument Rogers and Walker use to underscore the line-drawing problem is that lines cannot be identified even in incontrovertible cases such as infectious disease. They use acute cystitis (also known as acute uncomplicated urinary tract infection—UTI) to make their case. As there is no consensus as to what level of bacteriuria measured (in terms of the number of colony-forming units (cfu) of (E-coli) bacteria cultured from a specimen of urine) is required in order to establish the presence of disease, there is a line-drawing problem. As they tellingly point out «one of the strongest indicators of dysfunction, the bacterial count, is conventionally chosen, contested, and may be present with or without UTI» [74].

However, the bacterial count is a measure of dysfunction, but not the dysfunction itself. Admittedly, the bacterial count is a much stronger measure of dysfunction than BMI or blood pressure (which have been called indicators above), but one still has to show that the line-drawing problem relates to dysfunction and not to its measures. Hence, Rogers and Walker apparently confuse an indicative measure with dysfunction. As they point out themselves: «The presence of bacteria in the urine would seem to *indicate* that some protective function is not working properly» (my emphasis) [74].^1^

## Alternative Solutions to the Line-Drawing Problem

Hence, while Rogers and Walker have convincingly shown that the line between health and disease cannot be drawn on the level of indicators, processes, and measures, this does not amount to that «dysfunction cannot draw the line between health and disease.» The reason is that the relationship between indicators, processes, and measures on the one hand and functions on the other is unclear.

Note that Rogers and Walker’s conclusion may still be true. I have only argued that their arguments for the conclusion are not sound. We still have three alternatives:

(A) Dysfunction cannot provide a criterion of demarcation between health and disease.
(B) Dysfunction can provide a criterion of demarcation
(C) Other aspects of health and/or disease can provide criteria of demarcation between health and disease

Allow me therefore briefly to investigate some alternatives to address the line-drawing problems that have been less discussed in the literature. Three of these related to alternative B: One relates to biological evaluationism, another to alternative conceptions of function, and the third to the level of function. I will then return to the question of whether we look for the line in the wrong place, and particularly if it can be found in the relationship between disease and suffering (Alternative C).

### Drawing Lines in Biology

One alternative way to draw the line between health and disease is to claim that the evaluations involved in the assessment of dysfunction are purely biological (teleological and empirical) or non-evaluative. According to Boorse’s BST, function is the factual contribution to future survival and reproduction, and «normal functioning» is simply statistical normality within a particular reference class [5, 8]. Hence, it is the reduction of fitness in terms of survival and reproduction which is the normative source for drawing the line [78].

Hausman offers «friendly amendments» for a «revision of the BST» that relies on a goal-contribution view of functions, that links health to the efficiency of «part functioning,» and «that relies in part on the statistical distribution of capacities to distinguish healthy from pathological part functioning» [29]. In the FNC account (see Table 1) Schwartz combines criteria of frequency and negative consequences in order to draw the line between health and disease [78].

Contrary to Boorse and Hausman, Wakefield holds an evolutionary conception of dysfunction and adds the normative aspect of harmfulness (*harmful dysfunction*) [85, 86, 88]. Ananth also defends a naturalistic account of health and disease, based on an evolutionary concept of health that draws on concepts of biological function, species, and homeostasis [2] where a functional account of health and disease is based on a propensity concept of function.

In recent contributions, Griffiths and Matthewson defend an evolutionary («the selected effect») account of function [27] and combine it with the fitness aspects: «A mechanism can fail to perform the function for which it has been selected (due to mechanism failure or because of an abNormal environment) or biological fitness can be reduced (due to an inhospitable environment or the failure of a heuristic).» [55] Thereby they combine the backward-looking aspects of evolution (selected effect) and the forward-looking aspects (fitness) of biological normativity.

Yet others, such as Jon Lindstrøm, claim that «all diseases and dysfunctions must be characterized in teleological-evaluative terms. The relevant characterizing is however done at a useful level of explanation in empirical biology,…, nothing (medico-)ethical is necessarily implicit in bald bioevaluative practice. Being empirical in nature, questions of biological dysfunction can also yield to factual answers.» Moreover, «naturalists are quite capable of defining a concept of disease that only requires non-ethical evaluations of biological dysfunctions.»[51].

Another alternative is given by Thomas Schramme in his interpretation of Boorse’s conception of health and disease. According to Schramme, the line between health and disease can be drawn, but it is entirely theoretical. «We aim to establish general, ideally law-like, rules about when to call a certain condition pathological.» [76]. However, Schramme (and Boorse) may face with the opposite problem to Rogers and Walker, as a theoretical distinction between health and disease may be of scholarly interest, but have little relevance for practice. The clear theoretical line may be as irrelevant for practical line-drawing as the practical line-drawing problems may be for theoretical demarcations.

Hence, as I have tried to show, there still exist arguments that may be able to draw the line between health and disease based on the concept of dys-function (alternative B above). While I may think they are wrong (and agree with Rogers and Walker), I still need to show why they are so. However, that is the task for another article.

Let me now turn to the question of whether the problem of drawing of drawing the line between health and disease is not in the drawing, but in our (over-ambitious) conception of a line.

### Alternative Level of (Dys)Function

As aluded to, one approach to defend a function-based line between health and disease is to appeal to different levels of function. One can argue that while there is no line to be seen on one level of biological function, the line may be drawn on other levels (of function), for example on the overall activity level of the system [17, 69]. Hence, despite excellent work on the issue of function [27–29, 46, 49, 55, 75, 77, 78, 86, 87, 89], one may argue that more work could solve the problem.

One could, for example specify the level of function on the experiential level of the person. While it could be argued that this would remove the concept of dysfunction away from the pathophysiological or evolutionary level of BST and HDA, it would still provide a function-based way of drawing the line. Accordingly, how persons function in their daily life, may be a more appropriate level for drawing the line between health and disease than the (micro-)level biological dysfunction.^2^ As argued above, while many consider these and other types of functioning as value-laden concepts [35], I accept that others think that they are descriptive (and not normative) [51, 55].

The point here is therefore not to claim that the solution to the line-drawing problem can be found when defining (dys)function on a different level than the strictly biological. It is only to indicate that there are relevant alternatives that have not been sufficiently explored. The reason that I do not dive deeper into these alternatives is that I think that another line of argument may be more fruitful outside realm of dysfunction (B), i.e., in terms of manifest suffering (C).

## Drawing the Line at Manifest Suffering: Reconnecting Disease and Suffering

One reason for the recalcitrance of the line-drawing problem may be that we are looking for limits in the wrong place. It could be that functions are not appropriate for drawing the line between health and disease. Maybe the line has to be drawn in the realm of norms and not of nature [1, 25, 45, 48, 54, 71], or more precisely in the norms that make us classify nature? The reason why this question appears adequate is that the reason that we are interested in dysfunctions in the first place, is not the (dys)function as such, but the harm and negative experience that they are believed to produce. The reason why we are interested in the function of the heart and its cardiac output (CO) measures or troponin levels, is because of their connections to people’s experiences of what professionals call myocardial infarction. As succinctly stated by Canguilhem many years ago: pathology comes before physiology [11].

Accordingly, the reason why the limit between health and disease cannot be found in the realm of dysfunction (seen as a pure descriptive concept) is because the conception and definition of disease is derived from the realm of personal experience of pain and suffering. Dysfunction of the heart is not defined by its ability to pump blood (or to produce troponin, or murmurs), but because whether this specific level of pumping blood is experienced in specific ways and is harmful for the person. It is because a group of people experience a specific type of pain and suffering, that health professionals try to investigate them to find (a) identifying characteristics (symptoms), (b) signs and markers, as well as (c) related mechanisms, causes, or functions. It is the presence or absence of pain and suffering that make professionals group events and conditions with specific characteristics (a–c) and give them labels and define them as diseases. Hence, the source of the line is to be found in the realm of experience and prescription, and not in the realm of descriptions.

If this is correct, we should not look for the line between health and disease in the realm of biological functions, but for what instigates the definition of disease and such dysfunction in the first place, i.e., the in the realm of negative experience. Of course, not all sufferings qualify for disease. Only where health professionals have helpful categories, explanations, causal connections, and/or effective measures, is their attention warranted. Only those types of suffering that are related to something manifest (such as a tumor) and/or where health professionals can offer effective help [47]. However, it is the observed fatigue, loss of appetite, weight loss, and pains that make the health professional look for (signs of) altered functions, define these as dysfunctions, search for measures of treatment or prevention, and define it as a disease [34, 39].

Nonetheless, as we have become better at measuring features and functions, indicators and processes, we tend to make these characteristics define disease, and may come to decouple disease from its experiential origin [39]. This explains why Rogers and Walker cannot find any lines between health and disease in the indicators, processes, and measures [74]. These characteristics are too detached from what originally justified the definition of disease. They are only remotely related to suffering and death.

How then, can we demarcate health and disease based on the experience of suffering? How can we draw the line by focusing on what constitutes disease and what instigates the definition of and search for dysfunction in the first place? It is argued elsewhere that the line-drawing problem stems from the expansion of the concept of disease and from decoupling diagnosis (labelling) from experienced harm, such as pain, feeling tired, reduced abilities, nausea, and ultimate death [41]. Disease matters to people, but not mainly because we define or detect something as disease. It does so because it is closely connected to suffering. The closest connection is given by observable manifestations, i.e., palpable phenomena, such as painful tumors [39]. Again, not all suffering is due to disease, but all disease is (ultimately) related to suffering (in some way). If we have found indicators, processes, measures, or dysfunctions that cannot be connected to experienced suffering in any sense, then we do not have disease. Hence, the line can be drawn by whether what we detect (and/or can manipulate) is closely connected to suffering, where «closely» refers to what matters to people. When a certain indicator, process, or measure may forerun suffering, but where it is (prognostically) uncertain whether it does, we are not dealing with disease.

Hence, suffering can be a core concept for demarcation. However, this presupposes that we have a clear concept of suffering. This is not the place to enter the vast and vibrant debate on suffering [3, 18–21, 26, 31, 43, 53, 56, 59, 70, 81, 82, 91]. However, suffering is typically defined in terms of an experienced negative feeling, threats to human agency, and/or loss or threat to an individual’s value system and has physical, mental, social, and existential aspects [38]. While suffering is a personal and subjective experience [12] it is not incomprehensible to others [38]. As already pointed out, suffering is a necessary condition for disease, but it is not a sufficient condition. Only those types of suffering that can be conceptualized, detected, and treated or prevented by health professionals qualify for being disease.

Again, the point has not been to present a full-fledged theory of disease based on suffering but merely to indicate that there are still relevant unexplored alternative approaches to addressing the line-drawing problem. Nonetheless, there are some obvious candidates for suffering-based definitions of «disease» that emphasize suffering, e.g., the definition of malady: «A person has a malady if and only if he has a condition, other than his rational beliefs and desires, such that he is suffering or at increased risk of suffering, an evil (death, pain, disability), loss of freedom or opportunity, or loss of pleasure in the absence of a distinct sustaining cause.»[14] How well this and other pleasure-based concepts of disease solve the line-drawing problem very much depends on the line-drawing abilities of basic concepts such as «loss,» «freedom,» «opportunity,» «pleasure,» and risk.

This warrants a separate study [16, 23]. Here the point is that when looking for the line, we should look for it, not where the light is strong, but where the line is most likely lost.

## Discussion

The point in this article has not been to provide a complete theory of health and disease with clear-cut criteria for demarcation. Much more modest, it has been to analyze one of the most recent and comprehensive contributions to the field arguing that there is a fundamental line-drawing problem in the philosophy of medicine. I have tried to show that while Rogers and Walker’s arguments for a line-drawing problem between health and disease certainly are relevant for indicators, processes, and measures of dysfunction, they are not so for dysfunction or disease as such. Then I have tried to indicate that there are still alternative function-based approaches and available resources in the literature to draw the line between health and disease within naturalistic conceptions of health and disease. Although they may not solve the line-drawing problem, as they encounter many of the problems already discussed in the literature, there are good reasons to investigate them and hear them out [2, 27]. Lastly, I have tried to indicate that the reason why the line between health and disease cannot be found in the realm of indicators, processes, and measures of dysfunction is that these have become too decoupled from the defining features of dysfunction: suffering. Hence, we may be better equipped for drawing the line between health and disease in other places than so far have been sought. It may be particularly fruitful to return to the origin of medicine, with the primacy of the suffering of the person (and the experience of illness) [33, 34, 36]. Accordingly, we need to look for the line in other (and traditional) places, and the theory of malady is one such place [13, 14, 16, 22].

While I think that those who believe we can describe dysfunction scientifically (e.g., based on scientific descriptions of manifest suffering) have more to say, and should be listened to, I do think that we are forced into the normativist camp [35]. We should look for the line between health and disease not in nature but amongst our norms. A full elaboration of this warrants a separate article.

Obviously, including suffering into the conception disease (or dysfunction) appears to lead us back to square one. Deciding what kind of suffering that qualify for defining disease and how it is to be assessed is clearly no simple task. Moreover, determining which level of suffering that is significant or determining when certain characteristics are «strongly» connected to suffering are challenging (normative) issues. However, looking for the line in the appropriate place is at least a significant improvement. Moreover, defining suffering in terms of negative physical, mental, social, and/or existential experiences threatening to human agency or to persons’ value system in ways that are conceivable by others [38] can be helpful. So can defining it in terms of death, pain, disability, loss of freedom or opportunity, loss of pleasure [14] and actionability. That is, when the suffering experienced by the individual and recognized by others, can be conceptualized, detected, and treated or prevented by health professionals we may come closer to drawing the line between health and disease than when searching for indicators of dysfunctions.

Certainly, basing disease on its connection to suffering must face with a long list of objections. Firstly, it may be argued that some human disease states involve unconsciousness (coma), which does not involve bodily pain or suffering. However, suffering is not restricted to bodily experiences or hedonistic values. Suffering may also be mental, social, and existential. A person in coma has reduced agency, and can be said to suffer [38]. Secondly, it may be argued that some people may be unable to feel pain (e.g., congenital analgesia or syringomyelia) or lack self-reflection (as in mania), and hence not be suffering. Here one could argue that as long as the condition does not result in suffering (of any kind), it does not qualify as disease. However, in many such cases it does (in the long run). Thirdly, biologists apply the concept of disease across the whole spectrum of life, including to non-sentient animals and plants, who cannot suffer. Admittedly, there is an anthropocentric affinity in the suffering model. However, it does not exclude human responsibility for animals [38]. Fourthly, there are many bodily conditions where people are suffering, but that are not disease, such as pregnancy [57]. While some may argue that pregnant persons are not suffering (in an over-all sense of the word), I think there are reasons to question this argument. On the other hand, the premise that pregnancy is not a disease is a *petitio principii*. Pregnancy and childbirth get so much attention by the health care system exactly because it is so closely related to suffering.

Hence, there are certainly many counterarguments against using suffering as a defining concept for disease and a demarcation to health. However, it is not yet clear that the objections prevail. It is also relevant to notice that Rogers and Walker’s view of health and disease are not very far from mine. In a recent well-researched paper on overdiagnosis, they define (a overdiagnosis-related concept of) disease as «X is a disease_ODx_ iff there is dysfunction that has a significant probability of causing severe harm.» [73]. See also [90]. Hence, disease, and thereby its demarcation to health (in this specific context of overdiagnosis) is related on dysfunction and harm. While harm can be defined as different from suffering, they both can refer to first-person experience. Of course, including suffering or harm in the definition of disease poses challenges [57], and unlike Rogers and Walker, I think that suffering is implicit in the conception of dysfunction. Nonetheless, our perspectives are not far apart.

It is also important to notice that there is an inbuilt ambiguity in the line-drawing problem. There is a difference between a blurred boundary and a non-existent boundary. Both can cause line-drawing problems. While the latter is a warranted view, most of the debates have been on the former. E.g., Rogers and Walker think that the line-drawing problem is because the concept of disease is vague. Accordingly, this article has addressed the blurred boundary issue.

Accordingly, this article is not as much a critique of the line-drawing problem that Rogers and Walker draw our attention to, but to where it is located and where we should focus the effort in order to bring the field forward.

Moreover, using suffering as a criterion of demarcation may lead medicine back to its origin, i.e., helping suffering individuals, and hinder a wide range of challenges with modern medicine, such as unwarranted expansion of disease (pre-disease, risk-factors), overdiagnosis, overtreatment, and medicalization.

## Conclusion

This article does not provide a full-fledged theory of health and disease accounting for clear-cut borders between these concepts. However, by analyzing one of the most recent and comprehensive contributions to the field I have tried to show that we may be looking for lines in the wrong place. While arguments for a line-drawing problem between health and disease certainly are relevant for indicators, processes, and measures of dysfunction, they are not so for disease or dysfunction as such. Alternative approaches to dysfunction may still do the job of drawing the line between health and disease. We should be open for those.

However, I have tried to show that the reason why the line between health and disease cannot be found in the realm of indicators, processes, and measures of dysfunction is that these have become too decoupled from the defining features of dysfunction and disease: suffering. Accordingly, we should return to the origin of medicine, with the primacy of the suffering of the person in order to find fruitful sources of demarcation. The reason indicators are interesting, processes are relevant, and functions are classified as *dys-*functions, is because they are related to manifest suffering. Whether suffering can be described or is prescribed, may be debated. I have argued, though, that the sources of drawing the line between health and disease may be more fruitfully sought in the realm of suffering.

In sum, it may be more fruitful to use the suffering experienced by the individual and recognized by others, and that can be conceptualized, detected, and treated or prevented by health professionals, to draw the line between health and disease than using indicators of dysfunctions. Moreover, this can bring medicine back to its origin, i.e., helping suffering individuals, and hinder a wide range of challenges with modern medicine, such as unwarranted expansion of disease, overdiagnosis, overtreatment, and medicalization.

## Footnotes

1 The distinction between measures and indicators may seem subtle, and Rogers and Walker are not clear about these concepts. In this article it is assumed that a measure of a function is closer to the function than an indicator. For example, HbA1C is an indicator of glycemic control, while glycemic control is a measure of blood glucose regulation. In the quotes by Rogers and Walker on the UTI, they do not only point to the line-drawing problem, but also the problem of selecting which measures that count.

2 Correspondingly, the issue of variation and context can be taken into account: If a person experiences that (s)he is not able to function in the job as a teacher, combined with a family life with three children and three elderly and frail parents close by, then the level of cardiac output may indicate reduced cardiac function even though another person with exact the same (age, weight and) CO may be labelled as healthy.
